# Primary Malignant Melanoma of the Oral Cavity: A Retrospective Study From a Tertiary Care Centre of North India

**DOI:** 10.7759/cureus.32621

**Published:** 2022-12-17

**Authors:** Kavya Udyavar Raviraj, Sonali Mishra, Aishwarya Chandra, Anupa Khanal, Nishi Jha, Arvind Kumar, Ravi Hari Phulware, Ashok Singh, Prashant Durgapal, Prashant Joshi, Deepak Sundriyal, Sanjeev Kishore

**Affiliations:** 1 Department of Pathology and Laboratory Medicine, All India Institute of Medical Sciences (AIIMS) Rishikesh, Rishikesh, IND; 2 Department of Pathology and Laboratory Medicine, All India Institute of Medical Sciences (AIIMS) Mangalagiri, Mangalagiri, IND; 3 Department of Medical Oncology, All India Institute of Medical Sciences (AIIMS) Rishikesh, Rishikesh, IND

**Keywords:** mucosal melanoma, pathology, epidemiology, north india, rare, oral cavity, aggressive tumour

## Abstract

Background: Mucosal melanoma is a rare but aggressive tumor associated with a poor prognosis arising from pigmented cells called melanocytes. They are usually asymptomatic and present in an advanced stage. It has an aggressive clinical outcome and is proven to be of poor prognosis.

Materials and methods: This is a retrospective review of the computer database and clinical records at the All India Institute of Medical Sciences, Rishikesh, India. The data between 2018-2022 were reviewed for all small biopsy or excision specimen-proven cases of oral mucosal melanoma.

Results: The most common site of involvement in the head and neck region is the nasal cavity and paranasal sinuses. In this retrospective study from our institute, all three cases presented involved oral cavities. The median age of presentation was 51 years. Some literature specifies male preponderance. Our patients presented clinically with a black nodule in the oral cavity, which was increasing in size and associated with bleeding. A biopsy performed confirmed the diagnosis of melanoma based on the morphology and immunohistochemical profile of the tumor cells.

Conclusion: Surgical resection is the mainstay treatment, followed by radiation postoperatively to reduce local and regional recurrence. Mucosal melanoma has a poor prognosis, and the majority of patients develop incurable metastatic disease.

## Introduction

Melanomas are highly aggressive malignant neoplasms arising from melanocytes, accounting for 1.7% of the global cancer cases presenting clinically at advanced stages. In 1885, Lincoln et al. reported the first case of mucosal melanoma. The majority of cases of melanoma are of cutaneous origin. The extracutaneous presentation can involve uveal tissue, mucosa of the eye, the mucosal membrane of the oral cavity, and the gastrointestinal, respiratory, and genitourinary tracts. Mucosal melanomas are a rare and aggressive group of neoplasms compared to cutaneous counterparts accounting for 1.4% of all melanomas. Sino-nasal origin is now recognized as the most common site of primary occurrence of mucosal melanoma in the head and neck (66%), with the oral cavity representing 25% of cases [1,2].

Because of its hidden location and rich vascularization, mucosal melanomas usually present at a more advanced stage and are associated with a higher mortality rate than cutaneous melanoma. They can arise from any mucosal membrane, like the nasal cavity, sinuses, oral cavity, vulva, vagina, anorectum [3]. The most common site of mucosal melanoma is the head and neck region, which accounts for 40-50% of mucosal melanomas. Further, the mucosal lining of the nasal cavity and paranasal sinuses are the most common site in the head and neck region, followed by the oral cavity. In the nasal cavity, it manifests in the form of nasal obstruction. In the oral cavity, it manifests with a painless, irregular area of pigmentation within the oral mucosa [4].

Most mucosal melanomas are lentiginous type followed by superficial spreading and nodular type. They can be melanotic or amelanotic type. Morphology varies from epithelioid, plasmacytoid, mixed, and spindle with solid, organoid, and pagetoid patterns of arrangement [5].

It is challenging to differentiate primary from metastatic melanomas. However, there are molecular features to make it distinct from cutaneous melanomas. BRAFV600 is positive in cutaneous melanomas and is not found in a mucosal variety [6,7]. Mucosal melanomas show chromosomal aberrations like gain of 6p,8q, or 1q, and 15-22% may show overexpression of c-KIT [8,9].

Surgical resection is the mainstay treatment, followed by radiation postoperatively, to reduce local and regional recurrence. Mucosal melanoma has a poor prognosis, and most patients develop incurable metastatic disease. The five-year survival rate is 14% and is similar for all mucosal melanomas, regardless of where the cancer starts in the body [6].

In our study, we attempted to establish the correlation between clinical and pathological findings along with providing the data and epidemiology of the tumor occurrence in the foothills of the Himalayan region of North India.

## Materials and methods

**Figure 1:**
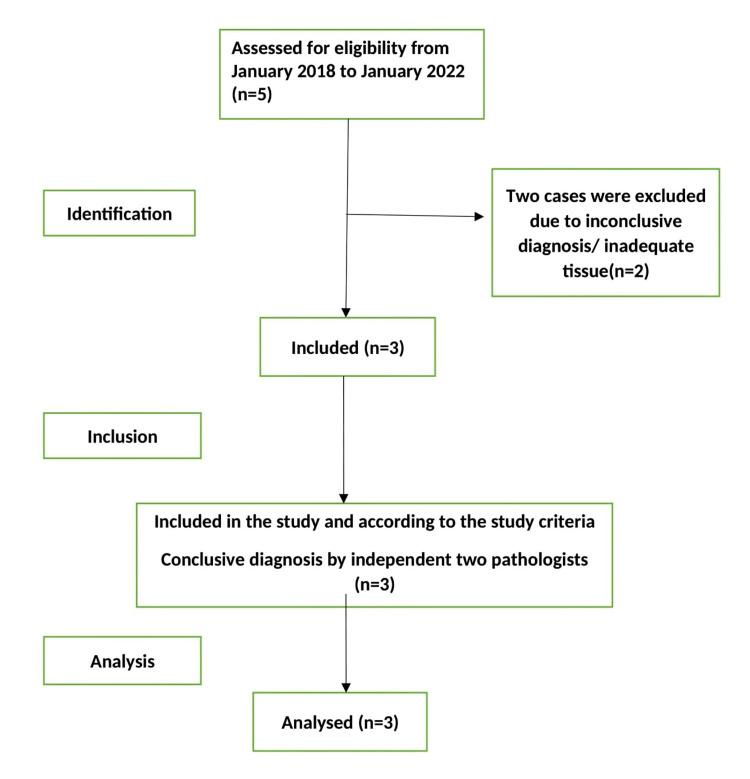
Study flow diagram

This is a cross-sectional study conducted at the Department of Pathology, All India Institute of Medical Sciences (AIIMS) Rishikesh, India, to ascertain the clinicopathological correlation and epidemiology of the disease in the Himalayan region of North India. Data was collected at one point in time. All hematoxylin and eosin stain (H&E) slides and immunohistochemistry (IHC) slides of oral mucosal melanoma cases were retrieved along with clinical details and requisition form, from the archives of the pathology department for the duration of four years, from January 2018 to January 2022. Only three mucosal melanoma cases were reported in that duration. All cases were reviewed by the two pathologists to confirm the diagnosis and subsequently included in the study. Only those cases included in the study have relevant clinical histories with adequate tissue in the biopsy sample. Cases with incomplete history, scanty tissue where IHC could not be performed, or inconclusive diagnoses due to incoherence between clinical details, microscopy, and immunohistochemistry were excluded (Figure 1).

## Results

In four years, 269 cases of oral cavity malignancy diagnosis were rendered. However, the diagnoses of 10 cases were reported as suspicious or inconclusive for malignancy. Excluding those cases, only 259 cases of oral cavity malignancy, which were confirmed on histopathology, were included in the study. Further review was done to sort out all the cases of mucosal melanoma, including suspicious and suggestive where IHC was lacking. Hence, only three cases qualified to be in the study due to having adequate tissue, relevant clinical history, and all the IHC panel. All the oral cavity malignancy cases, including confirmed cases, suspicious, suggestive, and inconclusive were further reviewed by the two pathologists to confirm the earlier diagnosis. Globally mucosal melanoma accounts for 1-2% of all melanomas, while in our institute, it accounts for 1.15% of cases over four years. Our study revealed that squamous cell carcinoma is the most common tumor of the oral cavity noted, followed by mucosal melanoma, epithelial myoepithelial malignancy of the minor salivary gland, and neuroendocrine tumor.

Case presentations

Case 1

**Figure 2:**
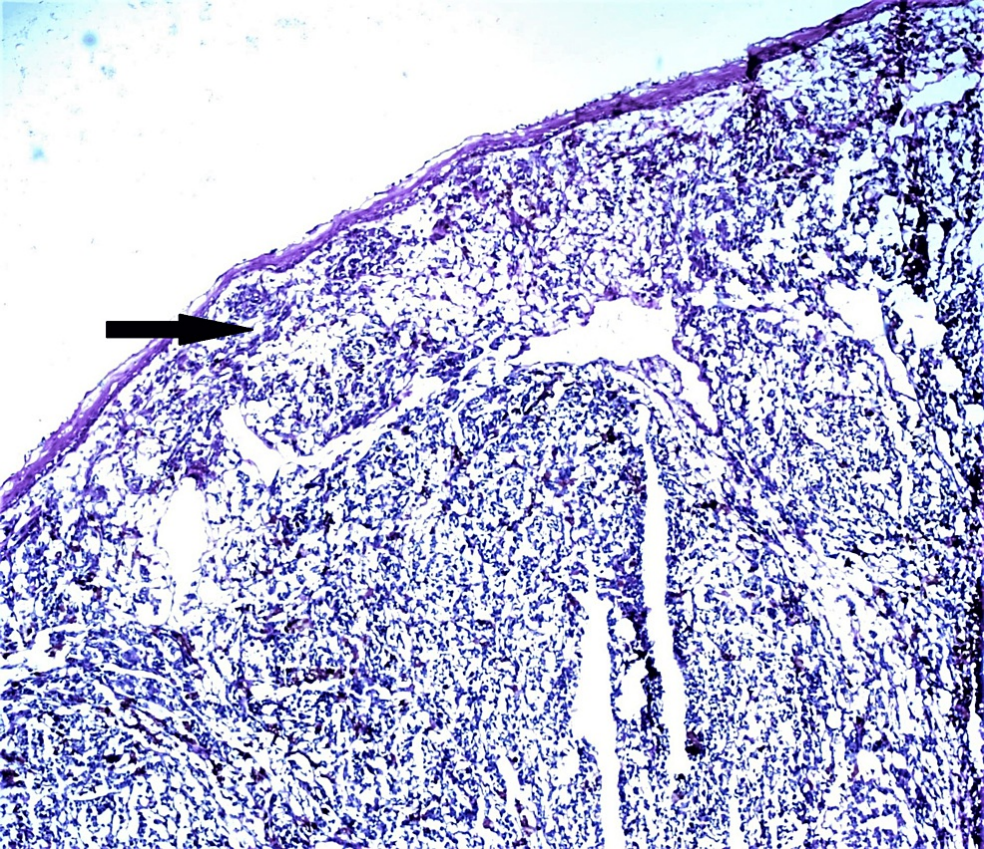
Tumour cells are monomorphic and disposed in sheet sand islands, infiltrating the pan dermis (Arrow) (H&E, 40X) H&E: hematoxylin and eosin stain

**Figure 3:**
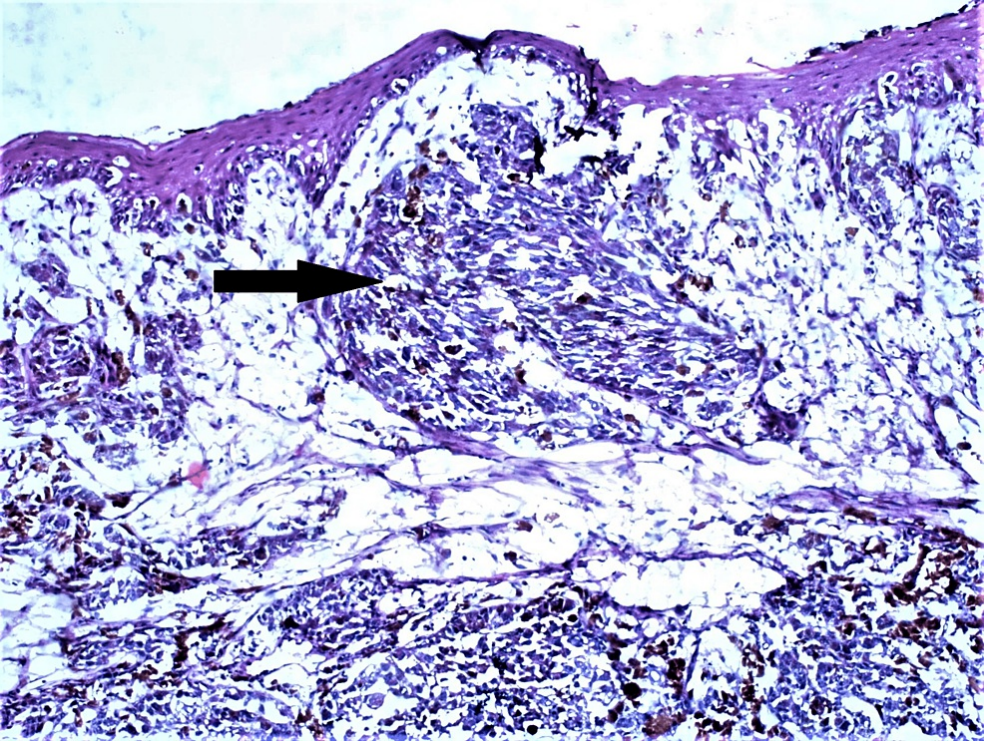
Lobules of tumour showing oval to spindled hyperchromatic nuclei with moderate amount of cytoplasm. Few of the cells exhibit dense melanin pigment (Arrow) (H&E,200X) H&E: hematoxylin and eosin stain

**Figure 4:**
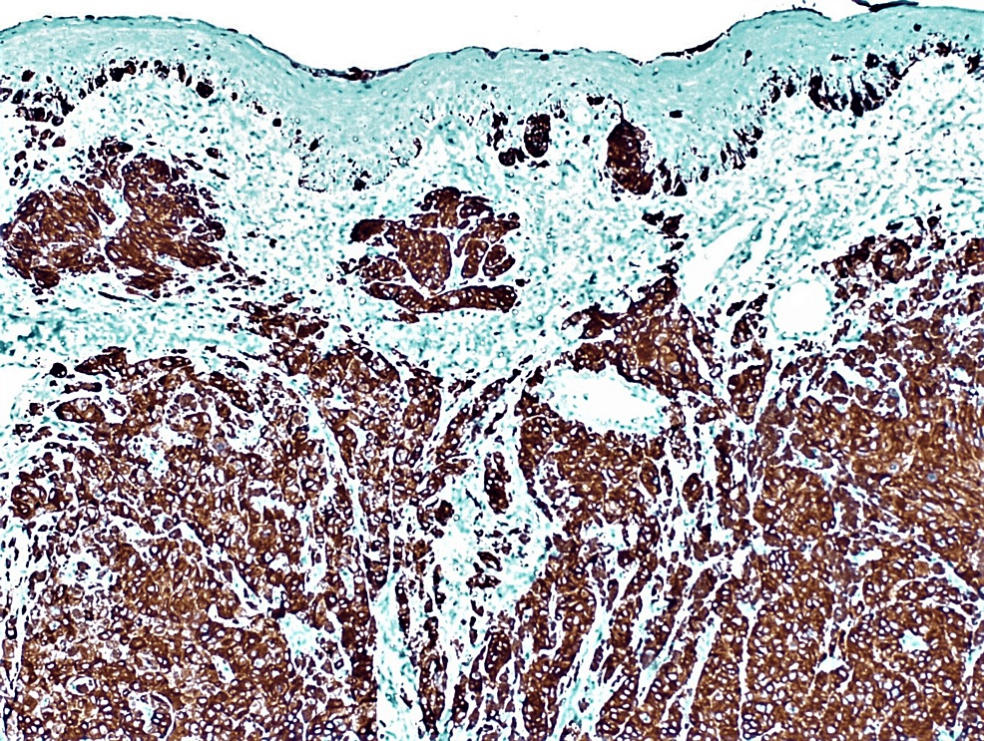
Immunohistochemistry of Melan-A antibody is positive in tumour cells (IHC,100X) IHC: immunohistochemistry

**Figure 5:**
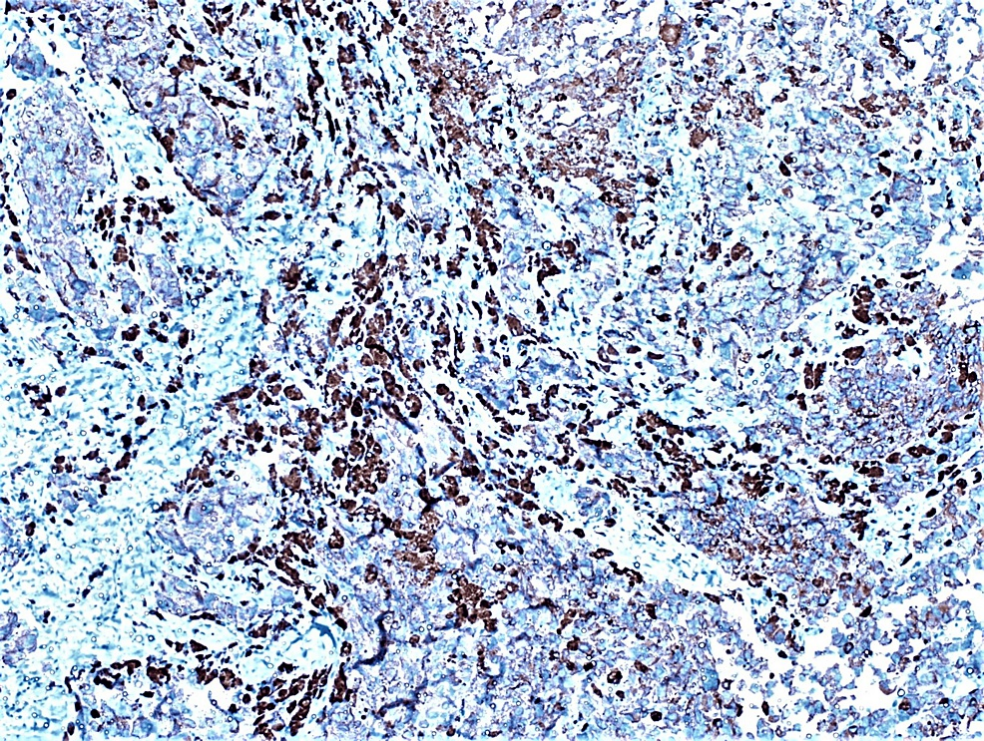
Immunohistochemistry of HMB-45 antibody is positive in tumour cells (IHC, 100X) IHC: immunohistochemistry; HMB-45: Human Melanoma Black-45

A 55-year-old male patient presented with a small black nodule in the lower jaw for two years and a history of occasional bleeding. The patient had no comorbidities or history of any tobacco or alcohol addiction. Clinico-investigative workup revealed regional lymph node metastasis, which was further confirmed by histopathology. An incisional biopsy of the lesion revealed tumor cells arranged in sheets, nests, and clusters with intracellular and extracellular brownish-black pigment. Tumor cells were pleomorphic and exhibit oval to spindled hyperchromatic nuclei with a high nucleo-cytoplasmic ratio (Figures 2, 3). However, on performing IHC, the tumor cells showed immunopositivity with Melan A and HMB45 (Figure 4, 5).

Thus, a diagnosis of melanoma was given. The patient underwent surgical resection that further confirmed the same tumor with the right lower alveolar site. The patient is now under follow-up.

Case 2

A 51-year-old male patient presented with brownish-black nodular swelling in the hard palate of the oral cavity. He mentioned having noticed the mass the last year and underwent an incisional biopsy from the site. The patient has been a chronic smoker for 20 years. Clinico-investigative workup did not reveal any regional or distant metastasis. The biopsy revealed the lesion to be melanoma, proven by IHC. The patient underwent a hemimandibulectomy for the same and is now under follow-up.

Case 3

 A 47-year-old male patient with known comorbidities of Type 2 diabetes mellitus presented with a black nodule in the oral cavity for a year, which was gradually increasing in size. Clinico-investigative workup revealed regional lymph node metastasis, which was further confirmed by histopathology. An incisional biopsy was performed, which showed a poorly differentiated carcinoma. The IHC profile showed positive tumor cells for S100, HMB45, and Melan A. Thus, a diagnosis of intramucosal melanoma was given. The patient was planned for surgical excision and was lost to follow-up.

The clinical presentations of the three cases are given in Table 1.

**Table 1:** Clinical Presentation of the cases

S/N	Age	Sex	site	Presenting symptoms	duration	Association with smoking/gutka	Treatment received	Metastasis
1.	55	Male	Lower alveolus	Black nodule with bleeding	2 years	Nil	Hemimandibulectomy and follow up	Regional lymph node infiltration present
2.	51	Male	Hard palate	Black nodule with ulceration	1 year	Chronic smoker	Hemimandibulectomy and follow up	absent
3.	47	Male	Lower alveolus	Tender black nodule	1 year	Nil	Biopsy and lost to follow up	Regional lymph node infiltration

## Discussion

Mucosal melanoma worldwide is most common among Blacks, Asians, and Hispanics. The literature says 9% of melanomas diagnosed in Blacks and Asians are of mucosal type [10]. There is not enough literature evidence regarding the incidences and prevalence across India. Melanomas are highly aggressive malignant neoplasms arising from melanocytes, accounting for 1.7% of the global cancer cases presenting clinically at advanced stages. Mucosal melanomas are a rare and aggressive group of neoplasms when compared to cutaneous counterparts, accounting for 1.4% of all melanomas. Sino-nasal origin is now recognized as the most common site of primary occurrence of mucosal melanoma in the head and neck (66%), with the oral cavity representing 25% of cases [1,2]. Forty percent of cases of oral mucosal melanoma are of amelanotic type [11]. The differential diagnoses to be ruled out in the case of amelanotic mucosal melanoma are large-cell lymphoma and poorly differentiated carcinoma are given in Table 2.

**Table 2:** Differential diagnosis of mucosal melanoma

S/N	Differentiating features	Amelanotic melanoma	Large cell lymphoma	Poorly differentiated carcinoma
1	Symptoms	Tender lesion over hard palate, erythematous flat lesion	Rapidly growing mass causing obstructive symptoms	Exophytic/ ulcerated mass
2	Microscopy	Epithelioid/fusiform/polymorphous cells infiltrating	Complete effacement of parent tissue, centroblastic/immunoblastic/anaplastic	Poorly differentiated tumor nests infiltration
3	On Immunohistochemistry	Positive for S100 (97%), HMB45 (85%), Melan A (71%)	Positive B cell for-CD20, CD79a, PAX8 Others: MUM1,,BCL6, CD10	Positive for CK5/CK6, p63, p16

Mucosal melanoma of the head and neck was first described in English literature by Lincoln in 1885 [12]. It was a case of melano sarcoma of the nose. In India, the largest study on mucosal melanoma was done by Gupta et al. in Tata Memorial Hospital, Mumbai; 42 cases of non-ocular mucosal melanoma of the head and neck were reviewed and followed between 1995-2003. The median age of presentation was 53 years [13]. Site distribution was found to be oral (55%), sinonasal (40%), and pharyngeal (5%). It was also concluded that age, sex, site of the primary tumor, tumor stage, and depth of infiltration did not affect the outcome of the disease significantly [14].

Most melanomas involve the head and neck area, most exposed to sunlight. However, the areas involved by mucosal melanomas are not ultraviolet-exposed areas, and hence literature has piling evidence for considering the melanocyte function in these areas to be of immunity [15]. The median age of presentation is around 61 years, with male preponderance.

We have documented three cases of oral mucosal melanoma in three consecutive years in AIIMS Rishikesh. The presenting features were subtle and were in an advanced stage. In our setup at AIIMS Rishikesh, about 259 cases of oral cavity malignancies have been reported between 2017-2022. Out of these, mucosal melanoma of the oral cavity contributed to 1.15% of the cases (Table 3).

**Table 3:** Intra-oral malignancy case distribution at AIIMS Rishikesh, India AIIMS: All India Institute of Medical Sciences

S/N	Histopathological Types	Total number of cases reported in 4 years, n = 259
1	Squamous cell carcinoma	253 (97.68%)
2	Epithelial myoepithelial malignancy of minor salivary gland	1 (0.38%)
3	Small round cell tumor	1 (0.38%)
4	Neuroendocrine tumor	1 (0.38%)
5	Mucosal melanoma	3 (1.15%)

Mucosal melanomas have poorer prognoses in comparison to their cutaneous counterparts. There are no established risk factors for mucosal melanomas [15], although irritants such as tobacco smoking and formaldehyde exposure are few associations considered (Table 4).

**Table 4:** Comparison of cutaneous and mucosal melanoma Table source: Seetharamu et al. [4], by permission of Oxford University Press

S.N.	Clinical Features	Cutaneous Melanoma	Mucosal Melanoma
1	Tissue of origin	Skin	Mucosal surfaces
2	Mean age at presentation	55 yrs	67 yrs
3	Staging	American Joint Committee on Cancer staging applicable	Not established
4	Presentation	Less than one-third present with advanced disease	>50% present with advanced disease
5	Amelanotic appearance	1.8%–8.1%	20%–25%
6	Risk factors	Sun exposure	Unknown
7	Race	White, 94%; black, 0.8%	White, 85%; black, 7%
8	Role of adjuvant radiation to the primary tumor	Generally no role	Radiation to the tumor bed generally recommended
9	Activating c-KIT mutations	<5%	15%–22%
10	BRAFV600E	50%–60%	Rare

Patient presentation is usually a locally advanced disease, such as a bleeding mass with ulceration or mucosal discoloration. Most oral mucosal melanomas involve mucosa covering maxilla. Half of the mucosal melanomas are the amelanotic type with no definite presentation [16]. They can also present as an already metastasized disease. All three of our patients presented with a black nodule associated with bleeding. There was no history of any addictive habits or any relevant findings in the family in two cases. However, one patient revealed being a chronic smoker (Table 1).

Microscopically, these tumors show cells that have large pleomorphic nuclei, prominent nucleoli, and nuclear pseudo-inclusion with intracellular melanin [17]. Cases without intracellular melanin are referred to as amelanotic melanoma. Diagnosis is further supported by immunohistochemistry with Melan A, S100, HMB45, tyrosinase, MART1, and MITF [17]. Vascular invasion, polymorphous tumor population, and necrosis seen on microscopy form poor prognostic factors.

In our cases, microscopy revealed sub-mucosal tissue infiltration by cells with marked pleomorphism arranged in trabeculae, clusters, nests, and small sheets. Cells had hyperchromatic nuclei with moderate eosinophilic cytoplasm. Intracellular and extracellular pigments were also identified. On immunohistochemistry, these cells were positive for HMB 45, Melan A, CD68, and S-100 with 80-90% Ki67, consistent with the literature [18]. 

Concerning the management of oral mucosal melanoma cases for locally advanced disease, the only curative treatment is complete surgical resection with negative resection margins. Radiotherapy following primary resection improves overall survival and rate of local disease control [19-20]. The first and the third cases underwent complete resection with negative resected margins. The second case was kept on follow-up due to the risk of sepsis because of uncontrolled diabetes. Complete resection was followed, achieving the desired diabetic control.

## Conclusions

Mucosal melanoma is a rare but aggressive malignancy that needs to be diagnosed as early as possible to avoid presentation in advanced stages. Hence, a clinician and pathologist must be well-versed in detecting this tumor. The clinical and pathological presentation of these malignancies is not familiar. Thus, it’s essential to report and study such cases so that the natural history of these tumors can be understood and the prognosis is improved by early diagnosis.

## Figures and Tables

**Figure 6:**
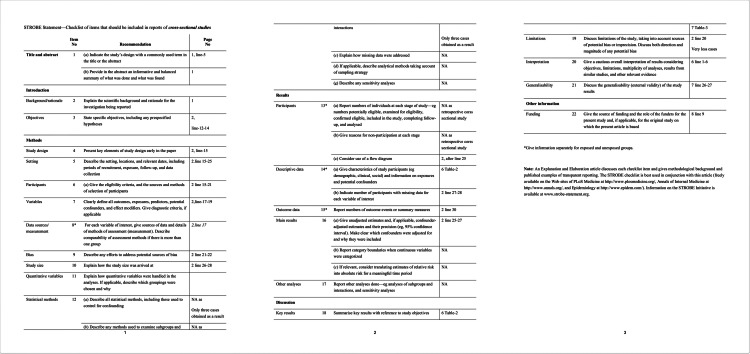
STROBE guidelines checklist STROBE: Strengthening the Reporting of Observational Studies in Epidemiology

## References

[1] Saginala K, Barsouk A, Aluru JS, Rawla P, Barsouk A (2021). Epidemiology of melanoma. Med Sci (Basel).

[2] McLaughlin CC, Wu XC, Jemal A, Martin HJ, Roche LM, Chen VW (2005). Incidence of noncutaneous melanomas in the U.S. Cancer.

[3] Thompson LD, Wieneke JA, Miettinen M (2003). Sinonasal tract and nasopharyngeal melanomas: a clinicopathologic study of 115 cases with a proposed staging system. Am J Surg Pathol.

[4] Seetharamu N, Ott PA, Pavlick AC (2010). Mucosal melanomas: a case-based review of the literature. Oncologist.

[5] Bishop KD, Olszewski AJ (2014). Epidemiology and survival outcomes of ocular and mucosal melanomas: a population-based analysis. Int J Cancer.

[6] Batsakis JG, Regezi JA, Solomon AR, Rice DH (1982). The pathology of head and neck tumors: mucosal melanomas, part 13. Head Neck Surg.

[7] Rimoldi D, Salvi S, Liénard D, Lejeune FJ, Speiser D, Zografos L, Cerottini JC (2003). Lack of BRAF mutations in uveal melanoma. Cancer Res.

[8] Davies H, Bignell GR, Cox C (2002). Mutations of the BRAF gene in human cancer. Nature.

[9] Curtin JA, Fridlyand J, Kageshita T (2005). Distinct sets of genetic alterations in melanoma. N Engl J Med.

[10] Cress RD, Holly EA (1997). Incidence of cutaneous melanoma among non-Hispanic whites, Hispanics, Asians, and blacks: an analysis of california cancer registry data, 1988-93. Cancer Causes Control.

[11] Paulo LF, Servato JP, Rosa RR (2015). Primary amelanotic mucosal melanoma of the oronasal region: report of two new cases and literature review. Oral Maxillofac Surg.

[12] Lincoln RP (1885). A case of melano-sarcoma of the nose cured by galvanocauterization. NY Med J.

[13] Gupta S, Vanderbilt CM, Cotzia P (2019). JAK2, PD-L1, and PD-L2 (9p24.1) amplification in metastatic mucosal and cutaneous melanomas with durable response to immunotherapy. Hum Pathol.

[14] Murthy V, Budrukkar A, Tejpal G (20101). Mucosal melanoma of the head and neck: Tata Memorial Hospital experience. Int J Otorhinolaryngol Head Neck Surg.

[15] Benlyazid A, Thariat J, Temam S (2010). Postoperative radiotherapy in head and neck mucosal melanoma: a GETTEC study. Arch Otolaryngol Head Neck Surg.

[16] Rose DS (1995). Nuclear pseudoinclusions in melanocytic naevi and melanomas. J Clin Pathol.

[17] Mihajlovic M, Vlajkovic S, Jovanovic P, Stefanovic V (2012). Primary mucosal melanomas: a comprehensive review. Int J Clin Exp Pathol.

[18] Notani K, Shindoh M, Yamazaki Y (2002). Amelanotic malignant melanomas of the oral mucosa. Br J Oral Maxillofac Surg.

[19] Nilsson PJ, Ragnarsson-Olding BK (2010). Importance of clear resection margins in anorectal malignant melanoma. Br J Surg.

[20] Kumar V, Vishnoi JR, Kori CG, Gupta S, Misra S, Akhtar N (2015). Primary malignant melanoma of oral cavity: a tertiary care center experience. Natl J Maxillofac Surg.

